# Advanced oxidation using modified enteromorpha algae-derived biochar for marine sediment dehydration

**DOI:** 10.3389/fchem.2025.1546240

**Published:** 2025-03-03

**Authors:** Lun Tan, Jian Zhang, Jiaoyang Du, Lianjie Guo, Hong Deng, Ying-Ying Gu

**Affiliations:** ^1^ National Marine Data and Information Service, Tianjin, China; ^2^ College of Chemistry and Chemical Engineering, China University of Petroleum (East China), Qingdao, China; ^3^ National Center of Ocean Standards and Metrology, Tianjin, China

**Keywords:** enteromorpha algae-derived biochar, persulfate, advanced oxidation process, dehydration, marine sediment

## Abstract

**Introduction:**

This study aims to enhance the dewatering performance of marine sediment using modified Enteromorpha algae-derived biochar to activate persulfate through an advanced oxidation process. Dehydration of marine dredged sediment is a challenging issue in the field of environmental remediation. Traditional dewatering methods are often inefficient due to the high water content, high Cl⁻ levels, and large volume of marine sediment.

**Methods:**

To address this, we developed an effective and environmentally friendly dewatering approach using biochar as a conditioning agent in combination with the strong oxidizing properties of persulfate. The biochar was prepared by pyrolyzing Enteromorpha algae at high temperatures and modified with FeSO_4_ solution to enhance its dewatering performance.

**Results:**

The results showed that under the optimal conditions of adding 4% of modified biochar and 1% of persulfate, the specific resistance to filtration (SRF) of sediment decreased by 73.4%, the yield of net solid (Y_N_) increased by 105%, and the dehydration amount increased by 118%.

**Discussion:**

The mechanism underlying the improved dehydration performance of marine sediment was revealed through the analysis of extracellular polymeric substances (EPS), zeta potential, compression coefficient changes, and microstructure analysis. It was found that Fe^2+^ compressed the double electric layer of sediment, while the activation of persulfate generated ·OH and SO_4_^−^· that effectively degraded EPS, thereby enhancing the dewatering performance. This research not only provides a new strategy for the sustainable treatment of marine sediment but also offers a theoretical basis for the resourceful utilization of algal biomass.

## 1 Introduction

The marine transportation industry has experienced unprecedented growth in recent years, particularly in the Jiaozhou Bay area of Qingdao, which has become a hub for significant shipping activities. However, this surge in maritime traffic has led to a substantial increase in the generation of marine sediment, posing a considerable challenge to both navigational safety and environmental health. The accumulation of marine sediment not only restricts the storage capacity of water bodies but also contributes to the deterioration of water quality, thereby disrupting the ecological balance of marine ecosystems (Zhuang et al., 2018; Xiao et al., 2019). Marine sediment, characterized by its high water content, high Cl^−^ levels, and large volume, presents significant hurdles for transportation and subsequent treatment processes (Cao et al., 2019). Traditional dewatering methods often prove to be inefficient, necessitating the development of innovative approaches to address these challenges. The dewatering of marine sediment is critical not only for the disposal of dredged materials but also for the recovery of valuable land resources and the prevention of environmental pollution (Sha et al., 2021).

In response to these issues, researchers have explored various conditioning agents to enhance the dewatering performance of marine sediment. Among these, biochar, a carbon-rich material produced from the pyrolysis of biomass, has emerged as a promising candidate due to its unique physicochemical properties, such as high porosity, large surface area, and a plethora of functional groups (Berde and Jolis, 2021; He et al., 2022). Recent advancements in biochar-based composites, particularly those modified with metal oxides (e.g., Fe³⁺, Mn^2^⁺), have shown remarkable efficiency in reducing specific resistance to filtration (SRF) and increasing net solid yield (Y_N_) (Guo et al., 2021; Li et al., 2020; Wang et al., 2023b). These composites leverage the high surface area and porous structure of biochar, combined with the catalytic properties of metal ions, to improve sediment dewatering. For instance, Fe³⁺-modified biochar has been reported to reduce SRF by up to 60% and increase Y_N_ by 80% in wastewater sludge treatment (Chen et al., 2022b). On the other hand, advanced oxidation technologies have been extensively utilized in sludge conditioning. These technologies generate free radicals with potent oxidizing properties that break down sludge extracellular polymeric substances (EPS), converting bound water into free water and thus enhancing the dewatering performance. The persulfate advanced oxidation process offers a more robust oxidation capability and longer lifespan than hydroxyl radicals (·OH) through the action of sulfate radicals (SO_4_^−^), effectively degrading EPS in marine sediments and improving their dewatering capabilities (Guo et al., 2021; Qi et al., 2011a; Fan et al., 2019). Recent studies have demonstrated that the combination of persulfate with biochar-based composites can further enhance dewatering performance. For example, Fe^2^⁺-modified biochar activated with persulfate generates reactive oxygen species (e.g., SO₄⁻· and ·OH), which disrupt the floc structure and convert bound water into free water, leading to significant improvements in SRF and Y_N_ (Li et al., 2022; Smith et al., 2019). The employment of biochar activated with persulfate represents a cost-effective and environmentally friendly approach that is currently a focal point of research (Wu et al., 2016).

Enteromorpha algae, commonly known as sea lettuce, is a green macroalgae that has been identified as a sustainable feedstock for biochar production (He et al., 2013; Alhashimi and Aktas, 2017; Wang et al., 2023a). In regions like Jiaozhou Bay, where Enteromorpha algae blooms have become a recurring environmental issue, the utilization of this algae for biochar production presents an opportunity to address both the sediment dewatering challenge and the management of algal blooms (Chen et al., 2017). The application of Enteromorpha algae-derived biochar in the dewatering of marine sediment has the potential to offer an eco-friendly and cost-effective solution while contributing to the circular economy. Enteromorpha algae-derived biochar provides unique advantages, such as high surface area and porous structure, and the ability to address algal bloom management by utilizing Enteromorpha algae as a raw material. Through modification with FeSO_4_, the Fe^2^⁺ loaded on the biochar surface not only compresses the double electric layer of sediment particles, reducing interparticle repulsive forces, but also acts as an activator for persulfate, generating highly oxidative ·OH and SO_4_⁻· radicals. These radicals effectively degrade EPS in the sediment, releasing bound water and significantly enhancing dewatering performance.

This study aims to investigate the fabrication of Enteromorpha algae-derived biochar and its application in enhancing the dewatering performance of marine sediment from Jiaozhou Bay. The biochar was prepared under different temperatures to optimize its properties for dewatering enhancement. The effects of Enteromorpha algae-derived biochar on the specific resistance to filtration (SRF), net solid yield (Y_N_), and the underlying mechanisms were thoroughly evaluated. Utilizing Enteromorpha algae to produce biochar, which serves as a catalyst for persulfate, embodies the concept of “waste to treat waste.” In this context, biochar catalysts were prepared from Enteromorpha algae and modified with ferrous sulfate, which acts as both a skeleton builder and an activator for persulfate to degrade EPS in sediments, thereby enhancing their dewatering performance. Optimal experimental conditions were established, and the mechanisms behind the improvement in marine sediment dewatering were analyzed by examining specific resistance (SRF), net solid yield (Y_N_), extracellular polymer (EPS), compression coefficient, zeta potential, and the microstructure of the dehydrated sediment. The findings of this research contribute to the body of knowledge on sustainable conditioning agents for marine sediment dewatering and provide insights into the environmental management of algal blooms.

## 2 Materials and methods

**FIGURE 1:**
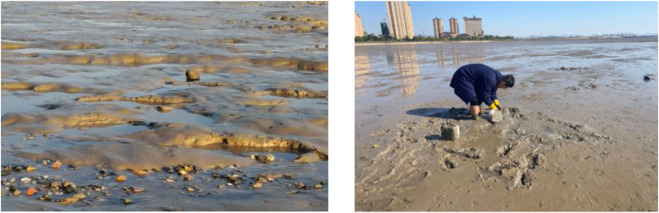
Sampling of marine sediment.

The marine sediment needed for the experiment is taken from Jiaozhou Bay in Qingdao, Shandong Province, China (Figure 1). It is transported back to the laboratory in a sealed polyethylene bucket and stored in a refrigerator at 4°C to maintain their properties until use. Before the dewatering experiments, the sediment samples were homogenized to ensure consistent moisture content. The Enteromorpha algae powder used was purchased from the Biological Technology Co., Ltd. of the Ocean University of China. The main parameters of the marine sediment are shown in Table 1.

**TABLE 1:** Main parameters of the marine sediment.

Water content (%)	pH	SRF(×10^11^ s^2^/g)	Y_N_(kg/m^2^s)	Electrical conductivity (mS/cm)	Cl^−^(mg/g)
60.0	8.7	6.99	0.0097	9.61	9.47

Note: SRF: specific resistance to filtration; Y_N_: net solid yield.

### 2.1 Preparation and modification of enteromorpha algae-derived biochar

Enteromorpha algae powder was used as the raw material. The dried Enteromorpha algae samples were pyrolyzed in a tubular furnace under a nitrogen atmosphere to prevent oxidation. One end of the tube furnace was connected to an alkaline solution to form a liquid seal. The Enteromorpha algae-derived biochar was prepared by heating it at different temperatures (500, 600, 700, and 800°C) to investigate the effect of temperature on biochar properties. Each sample was heated at a rate of 10 C/min and held at the final temperature for 2 h to ensure complete pyrolysis. The produced biochar was allowed to cool down under the same nitrogen atmosphere.

The Enteromorpha algae-derived biochar prepared at the optimal temperature was washed with 1 mol/L hydrochloric acid solution at a ratio of 1:5 (g:mL) and treated for 12 h. Then, it was centrifuged at 3,000 rpm/min for 30 min to remove the supernatant, and the sediment was collected. The sediment was washed with deionized water until the pH was neutral and then dried in an oven. The dried acid-washed Enteromorpha algae-derived biochar was soaked in ferrous sulfate solutions of 0.5, 1, 1.5, 2.0, and 2.5 mol/L at a ratio of 1:5 (g:mL), respectively. The treatment time was 12 h. After treatment, the mixture was centrifuged at 3,000 rpm/min to remove the supernatant, and the sediment was collected and dried in an oven. This resulted in Enteromorpha algae-derived biochar modified with different concentrations of ferrous sulfate. The physicochemical properties of the parepared Enteromorpha algae-derived biochar was listed in Table 2.

**TABLE 2:** Physicochemical properties of different Enteromorpha algae-derived biochar.

Characterized parameters		Enteromorpha algae-derived biochar				Acid-washed biochar (700°C)	2.5 mol/L FeSO_4_ modified biochar
		500°C	600°C	700°C	800°C		
Element content (wt%)	C	48.53	50.12	51.69	53.94	41.18	22.20
	O	45.40	44.32	42.57	40.56	53.45	55.79
	Cl	5.12	4.98	4.81	4.96	4.95	4.69
	Fe	0.22	0.28	0.32	0.18	0.18	16.46
	Others	0.73	0.30	0.61	0.36	0.24	0.86
pH		7.5	7.6	7.8	7.7	7.1	6.9
A_s_ (m^2^/g)		75.34	85.45	88.85	86.67	141.63	169.2

### 2.2 Dehydration experimental procedures

Marine sediment was mixed with different percentages (0%, 1%, 2%, 3%, 4%, 5%, and 6% by dry solid content) of the prepared biochar at different temperatures. The mixtures were stirred at 100 r/min for 5 min to ensure uniform distribution of the biochar within the sediment. Dewatering performance tests were conducted at a pressure of −0.01 MPa, and the SRF, Y_N_, and dewatering volume were measured to evaluate the dewatering performance.

To investigate the dehydration performance of marine sludge based on activated persulfate by modified Enteromorpha algae-derived biochar under different concentrations of ferrous sulfate, the modified Enteromorpha algae-derived biochar was added to the marine sediment and mechanically stirred for 15 min to ensure that it was thoroughly mixed with the sediment. Then, sodium persulfate was added to the marine sediment and stirred for another 15 min before allowing it to settle for 30 min. Next, 100 g of the treated marine sediment was placed in a Buchner funnel and subjected to a dewatering experiment under a pressure of −0.01 MPa. Concurrently, experiments adding solely modified Enteromorpha algae-derived biochar (4% DS) and solely sodium persulfate at dosages ranging from 0% to 1.2% DS were employed as control groups. The dewatering performance of the sludge was evaluated using the SRF, Y_N_, and cake moisture content. Additionally, changes in EPS, zeta potential, compression coefficient, Cl^−^ content, and microstructure of the sediment after dewatering were measured to analyze the dewatering mechanism.To ensure the accuracy of the experimental data, two parallel samples were set up for each experiment and the average value was taken.

### 2.3 Analysis method

The SRF, Y_N_ and the compression coefficient of the marine sediment was measured using a 60 mm Brinell funnel and a qualitative filter paper test device and calculated (Guo et al., 2020; Qi et al., 2011b; Thapa et al., 2009). The water content of marine sediment was calculated by gravimetric method. Conductivity meter to measure the conductivity of the marine sediment and Ion Chromatography (ThermoFischer, ICS-3000) was used to measure the Cl^−^ content; Zeta potential was analyzed by Zetasizer nanoanalyzer (Malvern, Zetasizer Nano ZS). EPR spectrometer (Bruker, EMX plus) was used to determine the species of free radicals produced by persulfate activated by modified Enteromorpha algae biochar. The microstructure of dehydrated Marine sediment cake was characterized by SEM (Hitachi, S4800).

## 3 Results and discussion

### 3.1 Optimization of preparation conditions for enteromorpha algae-derived biochar

**FIGURE 2:**
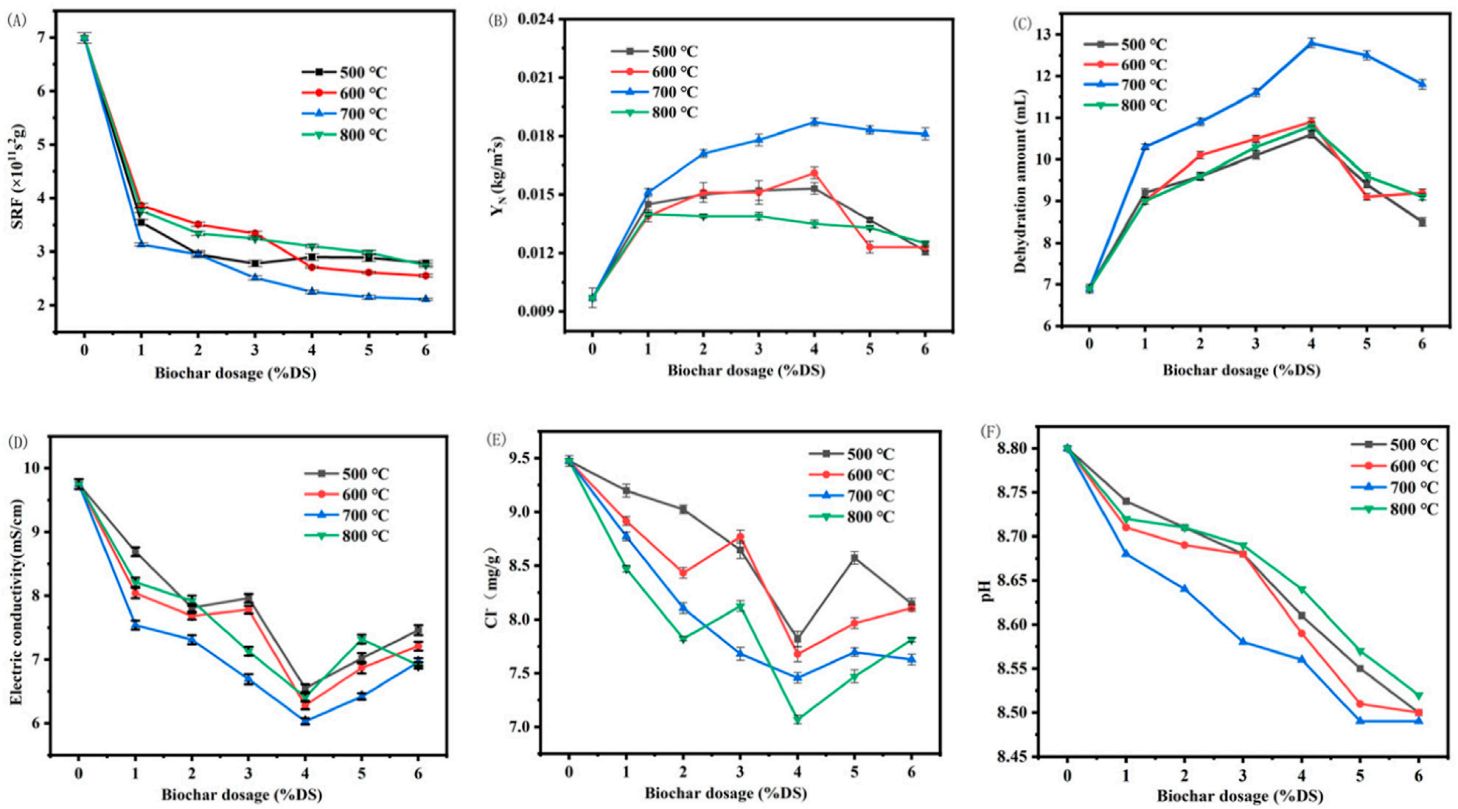
Effects of Enteromorpha algae-derived biochar dosage at different temperatures on the dehydration performance and parameters **(A)** SRF; **(B)** Y_N_; **(C)** dehyration amount; **(D)** electric conductivity; **(E)** Cl^−^ content; **(F)** pH of marine sediment. (SRF: Specific Resistance to Filtration; Y_N_: Net Solid Yield).

Our findings in Figure 2 reveal that the incorporation of Enteromorpha algae-derived biochar, pyrolyzed at distinct temperatures, markedly affects the dewatering efficacy of marine sediments. SRF measures the resistance of sediment to the filtration process. It is an indicator of how easily the sludge can be dewatered. A lower SRF value indicates that the sludge is easier to filter and dewater, which means it has better dewatering performance (Wang et al., 2023a). As can be seen from Figure 2A, the SRF of marine sediment after conditioning with Enteromorpha algae-derived biochar was significantly reduced. Y_N_ represents the amount of solids produced per unit area during the dewatering process. A higher Y_N_ value indicates that more solids are being recovered per unit area, which is desirable in wastewater treatment processes as it leads to a more concentrated solid product and improved dewatering efficiency. Figures 2B, C show that Y_N_ and dehydration amount were significantly increased. With the increase of Enteromorpha algae-derived biochar dosage, SRF decreased rapidly at first and then tended to be flat. With the increase of Enteromorpha algae-derived biochar, Y_N_ first sharply increased to the maximum value and then gradually decreased. The change trend of water removal and net solid production Y_N_ is basically the same.

The effects of Enteromorpha algae-derived biochar prepared at different temperatures on the dehydration performance of marine sediment increased first and then decreased with the increase of preparation temperature. Among them, the maximum Y_N_ was 0.0187 kg/m^2^s, the maximum water removal was 12.8 mL and the SRF was 2.25 × 10^11^ s^2^g when the preparation temperature was 700°C and the dosage of 4% DS. Compared with the original sediment, the SRF decreased by 66.4%, while Y_N_ and the water removal increased by 92.7% and 85.5%, respectively. The biochar produced at 700°C demonstrated the best dewatering performance can be attributed to its high surface area (88.85 m^2^/g) and functional groups, which enhance its adsorption capacity and catalytic activity. In contrast, biochar produced at 800°C exhibited lower performance due to excessive carbonization, which led to the collapse of pores and the loss of functional groups. These findings are consistent with previous studies, which suggest that moderate pyrolysis temperatures (600°C–700°C) are ideal for biochar production in dewatering applications (Guo et al., 2021; Li et al., 2020). Although SRF is still slightly reduced with the increase of biochar dosage, it may be due to the change of sediment content in the marine sediment, thus affecting SRF(Thapa et al., 2009). Therefore, Enteromorpha algae-derived biochar was prepared at 700°C, and the dosage of 4% DS had the best improvement on the dehydration performance of marine sediment.

Figures 2D, E illustrate that both the electrical conductivity and Cl^−^ content of the dewatered sediments initially decreased and then increased with escalating biochar dosages, closely tracking the variations in dewatering volumes. Notably, the minimum Cl^−^ content of 7.06 mg/g soil was attained at a 4% dry solid (DS) application of biochar pyrolyzed at 700°C, indicating a significant reduction in salt content. The minimal electrical conductivity and Cl^−^ content in the dewatered marine sediment at 700°C can be attributed to the optimal specific surface area of the Enteromorpha algae-derived biochar prepared at this temperature. The enhanced adsorptive capacity of the biochar at 700°C leads to a more efficient retention of ions, thereby reducing the Cl^−^ content and, consequently, the electrical conductivity of the dewatered sediment. This reduction could potentially expand the range of applications for the marine sediment post-treatment.

The pristine marine sediment exhibited alkaline properties, posing a risk of soil salinization during landfill disposal. Figure 2F demonstrates that the pH of the dewatered sediments decreased with the introduction of biochar prepared across a spectrum of temperatures. This decrease underscores enhanced dewatering performance and increased water expulsion, consequent to a diminished presence of OH^−^ within the sediment matrix. Strikingly, biochar derived at 700°C had the most pronounced effect on lowering the sediment’s pH, a critical parameter for its subsequent use in applications such as brick and ceramic pebble production. The decrease in pH observed in the dewatered marine sediment treated with biochar prepared at 700°C is likely due to the biochar’s increased adsorption of OH^−^ and its ability to neutralize the negative charges typically present in sediment particles. The larger surface area and reactive functional groups at this temperature facilitate the adsorption process, leading to a reduction in available OH^−^ and thus lowering the pH. Additionally, the biochar may release H^+^ or engage in reactions that consume OH^−^, further contributing to the acidification of the sediment.

### 3.2 Effect of activated sodium persulfate with modified enteromorpha

**FIGURE 3:**
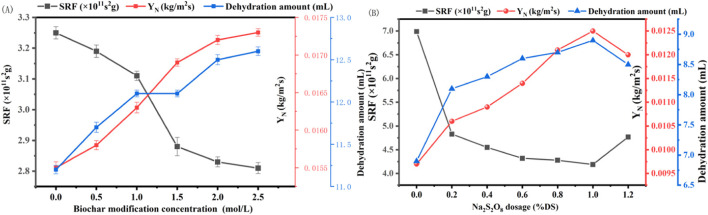
Effect of **(A)** modified Enteromorpha algae-derived biochar and **(B)** Na_2_S_2_O_8_ on the dewatering performance of marine sediment (SRF: Specific Resistance to Filtration; Y_N_: Net Solid Yield).

Our findings, as presented in Figure 3A, demonstrate that the dewatering performance improved progressively with the increase in the concentration of ferrous sulfate used for biochar modification. Notably, at a modification concentration of 2.5 mol/L, the biochar exhibited the lowest SRF of 2.81 × 10^−11^ s/g, a 13.5% reduction compared to the untreated sediment. Concurrently, the Y_N_ and water expulsion volume reached their peak values of 0.0173 kg/m^2^s and 12.6 mL, respectively, marking an 11.6% and 12.5% enhancement over the sediment conditioned with biochar prepared at 700°C. These improvements are ascribed to the electrostatic neutralization effect of the metal ions loaded on the biochar surface (Table 2), which reduces the repulsive forces between sediment particles, thereby promoting aggregation and enhancing the sediment’s settling and dewatering capabilities.

The modification of biochar with ferrous sulfate introduces metal ions that can interact with the negatively charged sediment particles, leading to a decrease in zeta potential and an increase in the stability of the sediment flocs. This, in turn, facilitates the release of bound water and improves the overall dewatering efficiency. The optimal modification concentration of 2.5 mol/L ferrous sulfate indicates a balance between the biochar’s adsorptive properties and its ability to neutralize the sediment’s negative charge, resulting in the most favorable conditions for dewatering.

The application of sodium persulfate as an amendment in marine sediment dewatering yielded intriguing results. As illustrated in Figure 3B, the SRF of the sediment decreased significantly with the addition of sodium persulfate, reaching an optimal condition at a dosage of 1% dry solid (DS). At this dosage, SRF was reduced by approximately 40%, from 6.99 × 10^11^ to 4.19 × 10^11^ s/g, while the volume of water expelled and the Y_N_ increased from 6.9 to 8.9 mL and from 0.0097 to 0.0125 kg/m^2^s, respectively. This enhancement in dewatering performance is attributed to the strong oxidizing property of sodium persulfate, which disrupts the floc structure of the sediment, converting bound water into free water, thus facilitating the removal of water from the sediment. However, an excess dosage beyond 1% DS led to a resurgence in SRF and a slight decrease in water expulsion and Y_N_, suggesting that higher concentrations may disrupt the microbial structure within the sediment, leading to an increase in viscosity and adversely affecting dewatering (Eaton, 2005).

**FIGURE 4:**
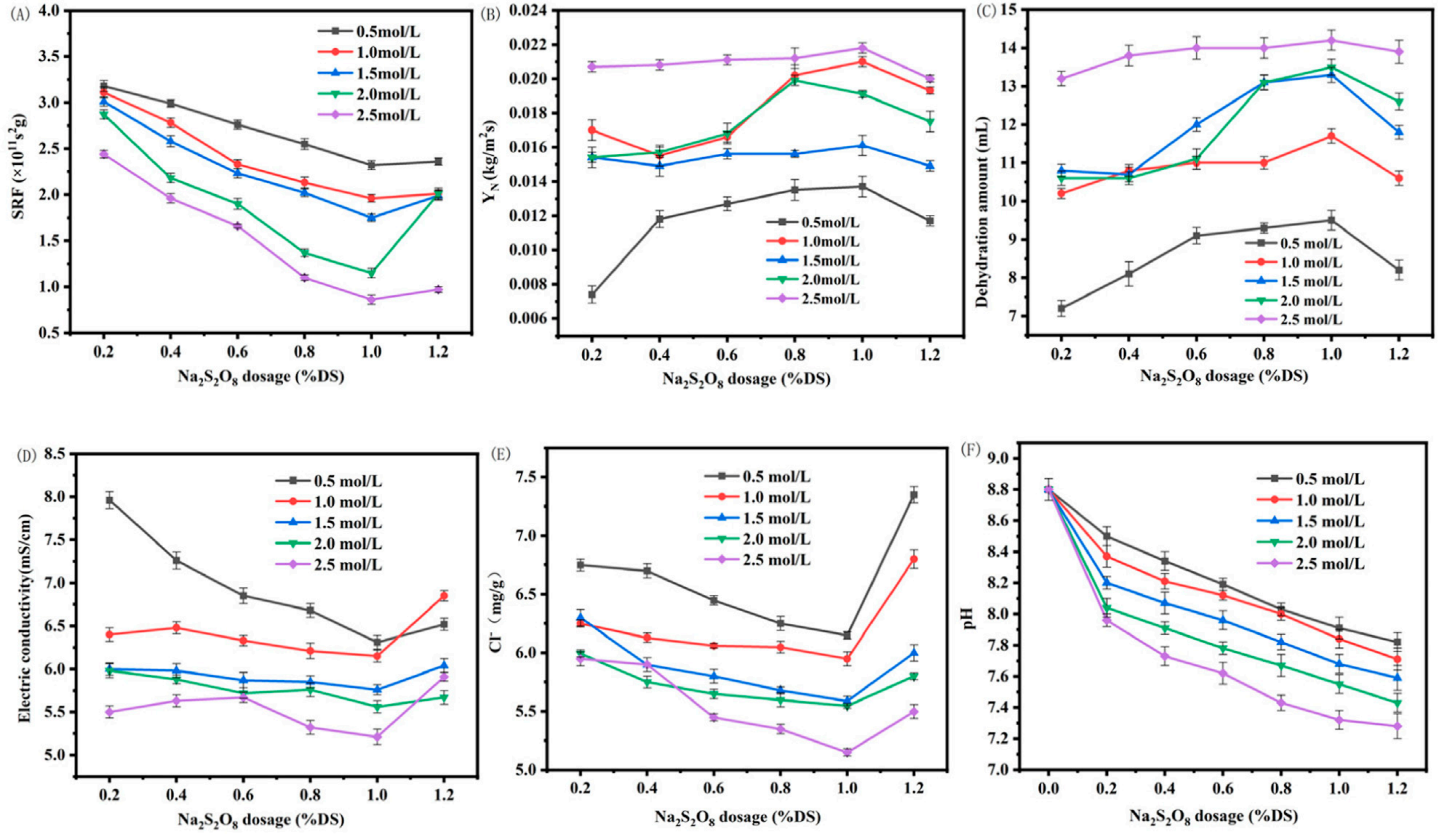
Effect of Na_2_S_2_O_8_ activation with modified biochar on the dehydration performance and parameters of marine sediment **(A)** SRF; **(B)** Y_N_; **(C)** dehyration amount; **(D)** electric conductivity; **(E)** Cl^−^ content; **(F)** pH of marine sediment. (SRF: Specific Resistance to Filtration; Y_N_: Net Solid Yield).

The combined treatment of marine sediment with modified Enteromorpha algae-derived biochar activated with sodium persulfate demonstrated a synergistic improvement in dewatering performance beyond that achieved with individual amendments. As shown in Figures 4A, B, the SRF decreased and the water expulsion and Y_N_ increased with the addition of sodium persulfate, reaching optimal conditions at a dosage of 1% DS with biochar modified with 2.5 mol/L ferrous sulfate. Under these conditions, SRF was minimized to 1.86 × 10^11^ s/g, Y_N_ peaked at 0.0218 kg/m^2^s, and the water expulsion volume reached 14.2 mL per 100 g of sediment as shown in Figure 4C. Compared to the original sediment, SRF was reduced by 73.4%, Y_N_ increased by 105.8%, and water expulsion volume increased by 118%. This significant enhancement is likely due to the generation of reactive oxygen species, such as SO_4_^−^· and ·OH, which are capable of breaking down EPS within the sediment as shown in Equations 1, 2 (Chen et al., 2010), releasing bound water and enhancing the sediment’s dewatering capacity. The presence of ferrous ions from the modified biochar further catalyzes the persulfate, leading to a more pronounced effect on dewatering performance (Qi et al., 2016).

$${\text{Fe}}^{2+}+S_{2}O_{8}^{2-}\to {\text{Fe}}^{3+}+{\text{SO}}_{4}^{-}\cdot +\;{\text{SO}}_{4}^{2-}$$ (1)

$${\text{SO}}_{4}^{-}\cdot +H_{2}O\to \cdot \text{OH}+{\text{SO}}_{4}^{2-}+H^{+}$$ (2)

The impact of modified Enteromorpha algae-derived biochar activated with varying concentrations of sodium persulfate on the electrical conductivity and chloride content of marine sediment post-dewatering was investigated. As depicted in Figures 4A, D, E significant reduction in both the electrical conductivity and chloride content was observed following the dewatering process. These reductions were attributed to the reaction between persulfate and trace metal ions present in the sediment, which enhanced the dewatering performance and consequently led to the removal of a substantial amount of ions along with the water, resulting in decreased conductivity and chloride levels.

With an increase in the dosage of sodium persulfate, an initial decrease followed by an increase in both electrical conductivity and chloride content was noted. This trend is indicative of the optimal dosage of persulfate, which in this case was found to be 1% DS. Beyond this point, the excessive persulfate seemed to disrupt the microbial structure within the sediment, leading to the release of cellular contents and an increase in sediment viscosity, adversely affecting dewatering performance.

Furthermore, the introduction of metal ions from the biochar, in conjunction with the persulfate, not only formed precipitates with inorganic ions in the sediment but also reduced the electrostatic attraction within the sediment, enhancing the dewatering performance and leading to lower electrical conductivity and chloride content (Chen, 2022). The most pronounced reduction was observed when the biochar was loaded with 2.5 mol/L ferrous sulfate and activated with 1% DS persulfate, with the lowest recorded values being 5.21 ms/cm for electrical conductivity and 5.1 mg/g for chloride content. Compared to the original sediment’s post-dewatering values of 9.75 ms/cm and 9.47 mg/g, these reductions represent a significant improvement of 91.2% and 44.98%, respectively. Such substantial decreases suggest a more favorable condition for subsequent utilization of the marine sediment.

The modification of marine sediment pH through the activation of modified Enteromorpha algae-derived biochar with different concentrations of sodium persulfate was also examined. As shown in Figure 4F, a notable decrease in pH values was observed following the dewatering process, with the pH trending towards neutrality. This decrease was more pronounced with increasing dosages of sodium persulfate and concentrations of ferrous sulfate in the biochar.

The pH reduction is primarily attributed to two reactions: the metal cations from the biochar react with OH^−^ to form precipitates (Equation 3), and the activation of persulfate by metal ions releases hydrogen ions, generating SO_4_^−^· radicals (Equation 4). Under alkaline conditions, these radicals react with hydroxide ions, further driving down the pH. The optimal condition, which resulted in the lowest pH values approaching neutrality, was achieved with 2.5 mol/L ferrous sulfate-modified biochar activated with 1.2% DS sodium persulfate. This neutralization is beneficial for the subsequent resourceful application of marine sediment, as extreme pH values can negatively impact the quality of products derived from sediment utilization.

$${\text{Fe}}^{2+}+{\text{OH}}^{-}\to \text{Fe}{\left(\text{OH}\right)}_{2}$$ (3)

$${SO}_{4}^{-\cdot }+{OH}^{-}\to \cdot OH+{SO}_{4}^{2-}$$ (4)

### 3.3 Mechanism of improving the dewatering performance of marine sediment

#### 3.3.1 Extracellular polymer (EPS)

Extracellular polymers (EPS) in marine sediment significantly influence the dewatering process. EPS constitutes an essential component of the sediment floc, with marine sediment particles being enveloped by EPS. EPS provides necessary nutrients to the cells, as it contains a plethora of hydrophilic oxygen-containing functional groups, such as -CONH- and -COO-. These groups hinder the flow between influent and effluent, impeding the release of internal water, thereby making the removal of bound water more challenging. The primary organic substances within EPS are polysaccharides (PS) and proteins (PN), the latter of which contains acidic amino acids, endowing it with a negative charge (Zhang et al., 2019). The presence of anions such as phosphate and carbonate in EPS further confirms its negative charge (Rong et al., 2021). Investigating the changes in PN and PS during the dewatering process aids in understanding that the presence of EPS is one of the fundamental reasons for the difficulty in dewatering marine sediment (Li et al., 2020). EPS can be categorized based on its existing form into soluble EPS (S-EPS) and bound EPS. The bound EPS is further divided into loosely bound EPS (LB-EPS) and tightly bound EPS (TB-EPS). The initial contents of PN and PS in the three EPS components (S-EPS, LB-EPS, and TB-EPS) of the marine sediment used in the experiment were 6.07, 6.29, and 10.25 mg/gVSS, and 0.06, 0.252, and 0.105 mg/gVSS, respectively.

**FIGURE 5:**
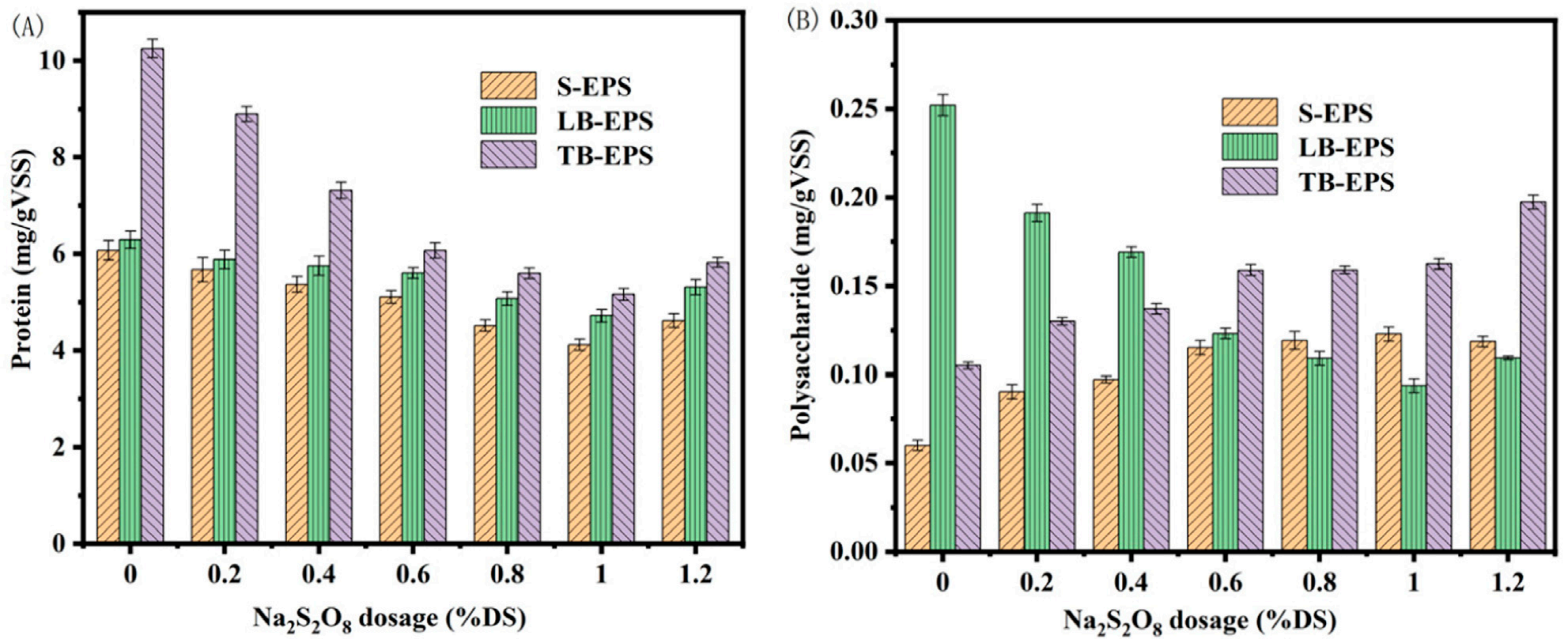
Changes of extracellular polymer in marine sediment with Na_2_S_2_O_8_ activated by modified biochar **(A)** protein; **(B)** polysaccharides.

Figure 5 illustrates the alterations in EPS upon activation with different concentrations of sodium persulfate using biochar derived from Enteromorpha algae and prepared at 700°C with a 2.5 mol/L ferrous sulfate solution. As the sodium persulfate concentration increased from 0.2% to 1.0% DS, a continuous decline in protein content was observed. At a 1.0% DS dosage, protein levels dropped to 4.12, 4.71, and 5.18 mg/gVSS, indicating the disruption of marine sediment EPS. The protein content gradually increased with further addition of sodium persulfate, mirroring the SRF changes post-amendment with modified biochar. S-EPS and TB-EPS polysaccharides increased with sodium persulfate dosage, while LB-EPS polysaccharides decreased, eventually stabilizing. The escalating concentration of sodium persulfate generates more SO_4_^−^·, leading to greater EPS damage within the sediment. The continuous reduction in protein content and the concurrent improvement in sediment dewatering performance suggest that proteins are detrimental to dewatering, with the most significant loss occurring in TB-EPS, indicating that effective degradation of TB-EPS substantially enhances sludge dewatering (Sheng et al., 2010). Apart from the decrease in LB-EPS, the other two EPS components gradually increased with sodium persulfate dosage changes. The reduction in LB-EPS polysaccharide content was attributed to the transformation of LB-EPS into S-EPS and TB-EPS under the influence of SO_4_^−^·. This finding aligns with the positive correlation between sludge dewatering performance and the concentration of organic matter in S-EPS, but a negative correlation with the biopolymer content in LB-EPS.

#### 3.3.2 Compression coefficient

**FIGURE 6:**
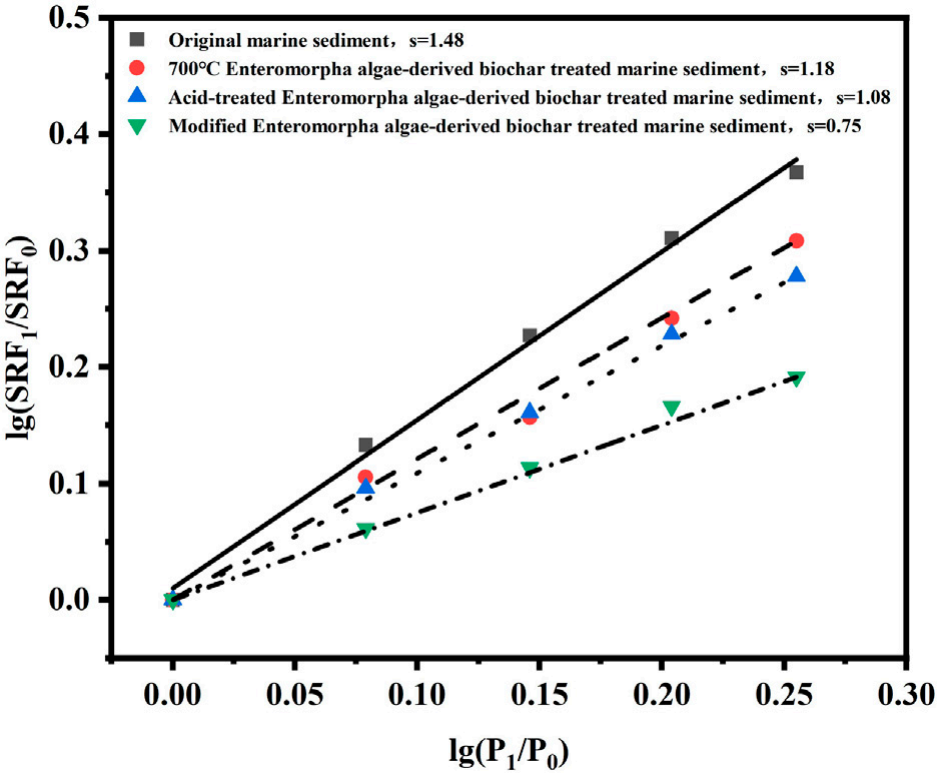
Compression coefficients of different treatment methods.

The compressibility coefficients of marine sediment under various conditioning regimens were determined to evaluate the impact of different amendments on the dewatering performance. Initially, the compressibility coefficient of untreated marine sediment was recorded as 1.48 as shown in Figure 6, indicating a high compressibility and a propensity for deformation during dewatering, which can impede the efficient removal of water. Upon the introduction of Enteromorpha algae-derived biochar prepared at 700°C, the compressibility coefficient was reduced to 1.18. This reduction suggests that the biochar amendment enhances the sediment structure, thereby decreasing its compressibility and improving dewatering efficiency. Further enhancement was observed when the sediment was conditioned with acid-treated Enteromorpha algae-derived biochar, as the compressibility coefficient decreased to 1.08. The acid treatment likely increases the biochar’s surface area and pore structure, augmenting its interaction with the sediment and further reducing its compressibility. Most notably, the application of modified Enteromorpha algae-derived biochar activated with sodium persulfate resulted in a significant decrease in the compressibility coefficient to 0.75. This substantial reduction indicates that the combined use of modified biochar and persulfate has a synergistic effect on sediment structure improvement (Zhou et al., 2018). The persulfate’s strong oxidizing nature is believed to disrupt the organic matter within the sediment, while the modified biochar provides a stable framework, together leading to a marked reduction in sediment compressibility. The compressibility coefficient of marine sediment decreases with the optimization of conditioning methods, indicating that these amendments effectively improve sediment structure and reduce its compressibility during dewatering, thereby enhancing dewatering efficiency. The combined use of modified Enteromorpha algae-derived biochar and activated sodium persulfate stands out as the most effective conditioning approach, significantly lowering the compressibility coefficient and offering a promising strategy for marine sediment dewatering.

#### 3.3.3 Zeta potential

**FIGURE 7:**
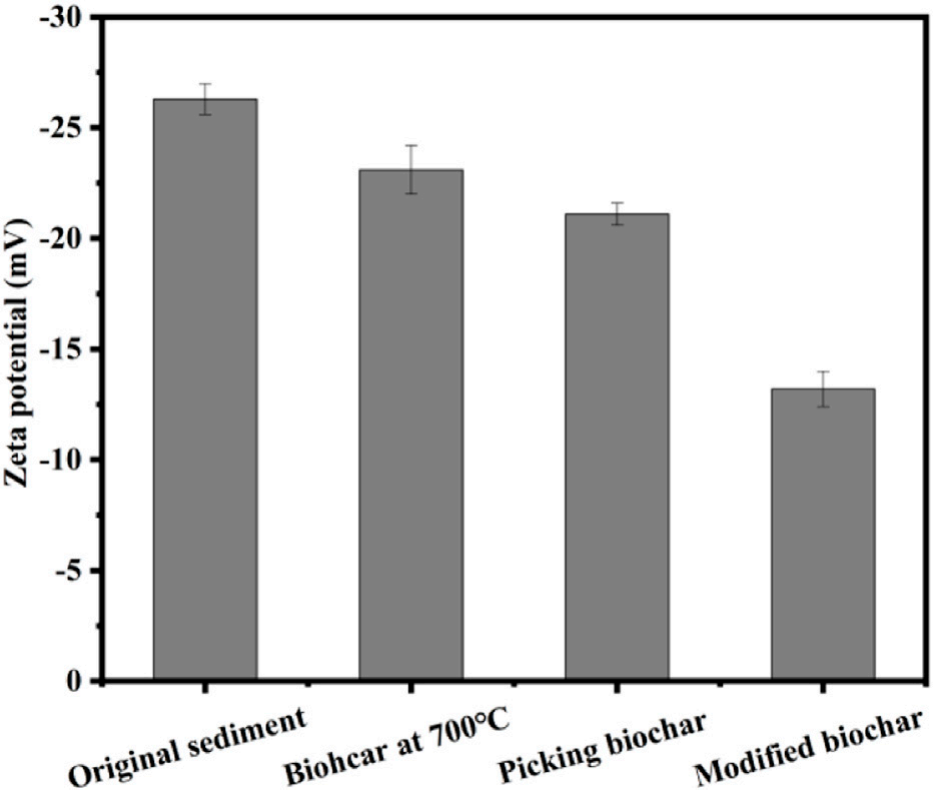
Zeta potential values of sediment with different treatments.

Figure 7 illustrates the zeta potential of the original marine sediment, Enteromorpha algae-derived biochar prepared at 700°C, acidified biochar added at 700°C, and Enteromorpha algae biochar activated with sodium persulfate by incorporating Enteromorpha algae-derived biochar modified at 2.5 mol/L FeSO_4_. As observed in Figure 7, the zeta potential of the marine sediment following activation with sodium persulfate by modified Enteromorpha algae-derived biochar is markedly reduced compared to other treatment methods, reaching −13.2 mV. This phenomenon primarily occurs because the modified Enteromorpha algae-derived biochar loads a significant amount of Fe^2+^ on its surface (Table 2), which neutralizes the negative ions within the marine sediment system (Guo and Zhou, 2020). This neutralization significantly reduces the zeta potential and disrupts the colloidal stability of the marine sediment through charge neutralization. Consequently, the repulsive forces between sediment particles are diminished, facilitating the aggregation of floc particles and enhancing the removal of moisture from the marine sediment. These effects are instrumental in the improved dewatering performance achieved with the activation of sodium persulfate by modified Enteromorpha algae-derived biochar (Guo et al., 2020).

#### 3.3.4 EPR analysis

**FIGURE 8:**
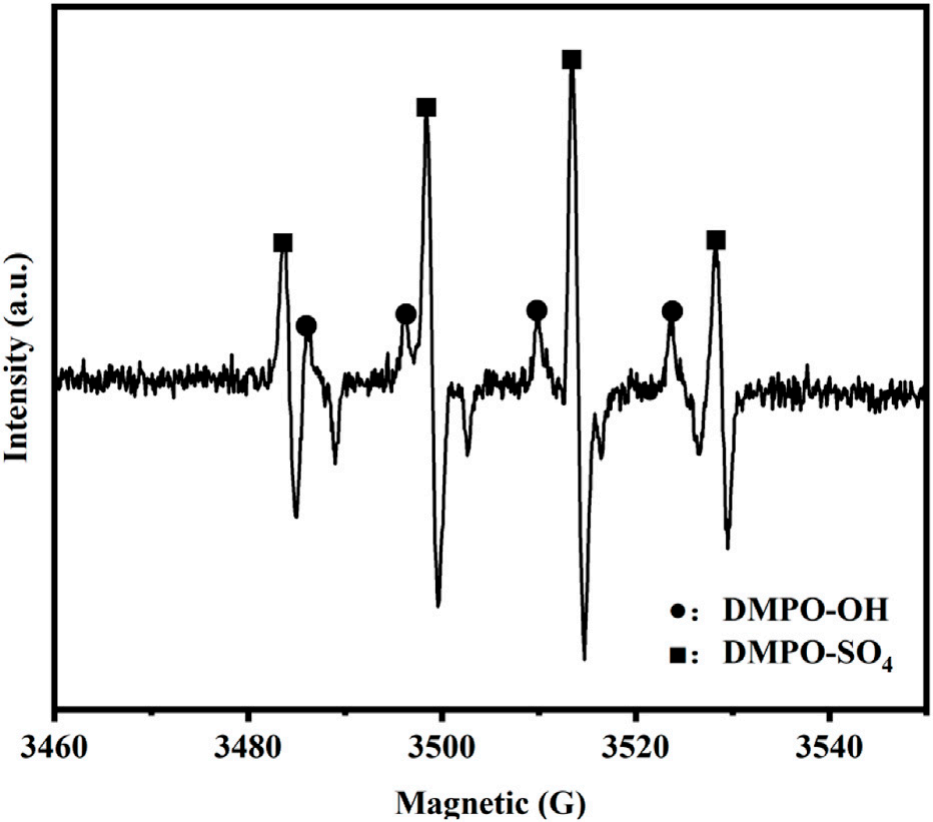
EPR analysis following the activation of Na_2_S_2_O_8_ using modified biochar.

Figure 8 presents the EPR analysis following the activation of sodium persulfate using modified Enteromorpha algae-derived biochar. The presence of ·OH and SO_4_^−^· in the activated system, as indicated by the DMPO-OH and DMPO-SO4 signals, suggests that their interaction can enhance the oxidative performance of the system (Liu et al., 2016). The Fe^2+^ loaded on the surface of the modified Enteromorpha algae biochar reacts with S_2_O_8_^2-^ from sodium persulfate via reaction (1), generating SO_4_^−^. Subsequently, SO_4_^−^· reacts with water to produce ·OH through reaction (2). Within the system, the presence of ·OH and SO_4_^−^ leads to the degradation of EPS in marine sediment, liberating more bound water and consequently improving the sediment’s dehydration performance.

#### 3.3.5 Microstructure analysis

**FIGURE 9:**
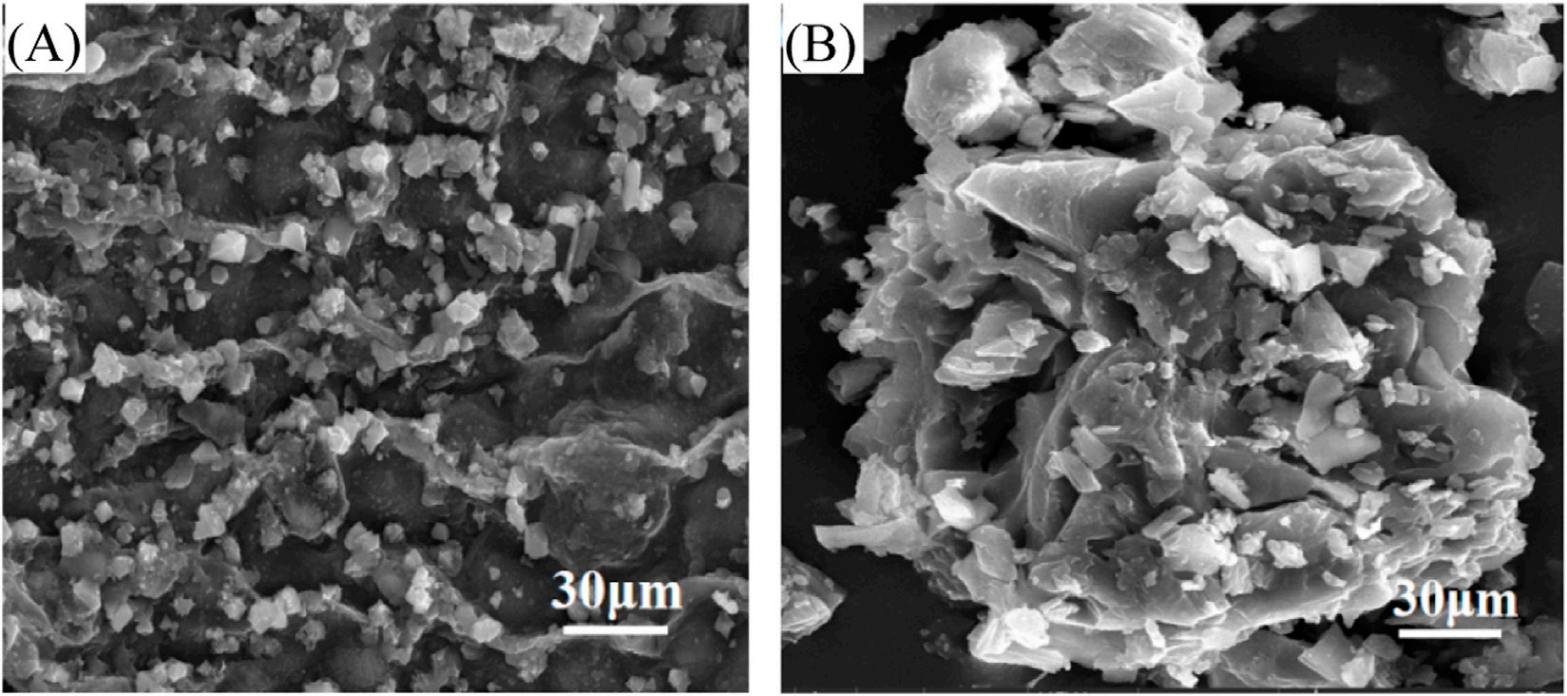
Surface structure of Enteromorpha algae-derived biochar before and after modification. **(A)** Original biochar; **(B)** FeSO_4_-modified biochar.

Figure 9 provide SEM characterization of the Enteromorpha algae-derived biochar prepared at 700°C and the biochar modified with 2.5 mol/L FeSO_4_. The SEM image of the biochar prepared at 700°C reveals a surface covered with fine particulate matter, indicative of the raw material’s inherent structure. These particles are likely remnants of the carbonization process, which contributes to the biochar’s porous and adsorptive properties. The presence of such particulates suggests a complex surface area that could potentially provide multiple sites for interaction with sediment components during the dewatering process. In contrast, the SEM image of the biochar modified with 2.5 mol/L FeSO_4_ displays a markedly different surface morphology. The modification results in a dense coverage of particulates on the biochar surface (Kurade et al., 2016), which is attributed to the FeSO_4_ treatment. These particles are predominantly composed of Fe^2+^ that have been adsorbed onto the biochar’s surface, leading to the formation of a rough, granular texture. This modification significantly enhances the biochar’s surface area and its capacity to interact with and neutralize the negative charges on sediment particles, thereby improving the sediment’s dewatering properties. The SEM results indicate that the FeSO_4_ modification introduces a substantial change in the biochar’s surface characteristics. The formation of a granular layer on the biochar surface post-modification suggests an increased ability to bind with and flocculate sediment particles. This is attributed to the Fe^2+^ ions, which are known to neutralize negative charges commonly found on the surfaces of sediment particles, thus facilitating aggregation and water release.

**FIGURE 10:**
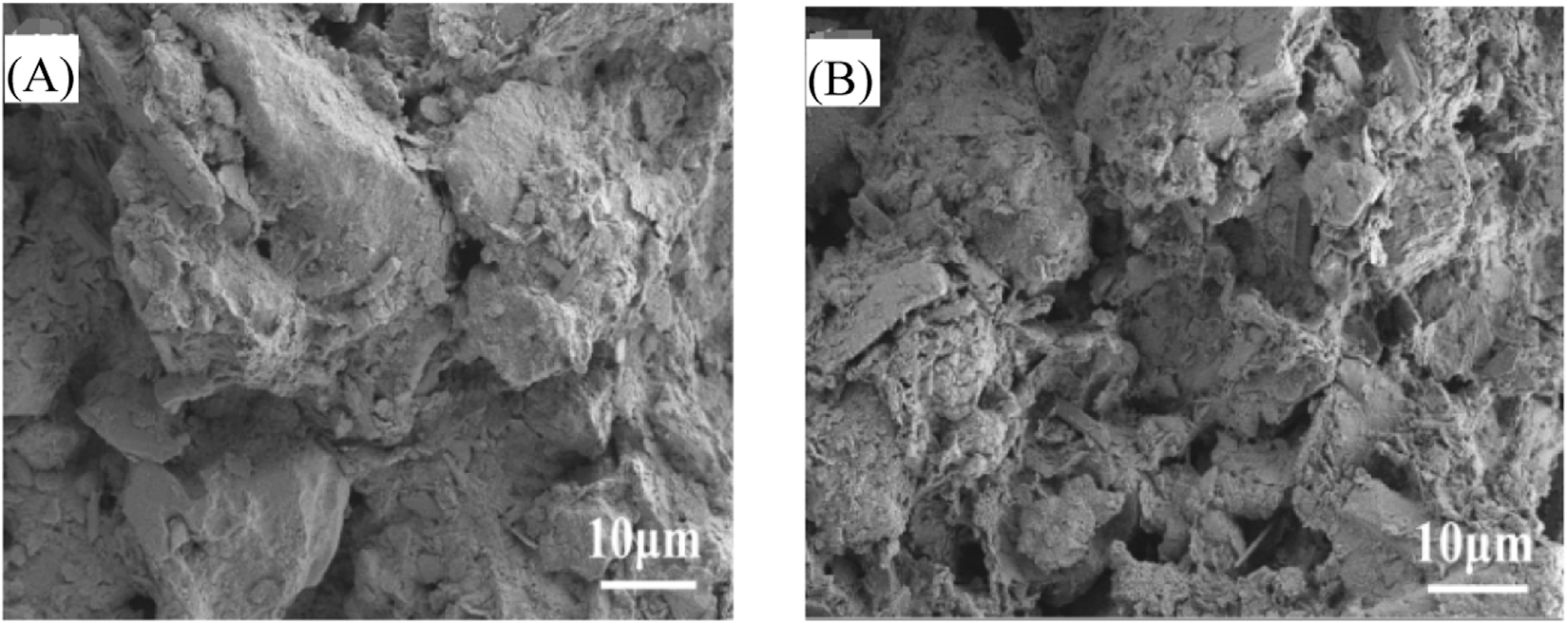
Microstructure of marine sediment after different treatment methods. **(A)** original marine sediment; **(B)** modified biochar activated Na_2_S_2_O_8_ treatment.

SEM was utilized to examine the microstructure of the sludge cake, as depicted in Figure 10. Figure 10A illustrates the microstructure of the original marine sediment at 10 μm. Figure 10B presents the microstructure of the marine sediment after the addition of modified Enteromorpha algae-derived biochar activated with sodium persulfate, also at 10 μm. It is observable that the original marine sediment cake possesses a compact, plate-like structure with low porosity, which is detrimental to the removal of water from the cake, indicating poor dewatering performance of the original marine sediment. Following treatment with modified Enteromorpha algae-derived biochar activated with sodium persulfate, there is a significant increase in the porosity of the marine sediment, with the biochar playing a skeletal role within the sediment (Chen et al., 2010). Additionally, a multitude of small pores appeared within the marine sediment cake. The cake’s surface became more fragmented, with an increased presence of fine particulate matter, rendering the marine sediment more loosely packed, which aids in the removal of water from the sediment (Guo et al., 2020). This further confirms that the activation of sodium persulfate by Enteromorpha algae-derived biochar contributes to enhanced dewatering performance.

The experimental results of this study demonstrate that the synergistic effect of Fe^2^⁺-modified biochar and persulfate significantly improves the dewatering performance of marine sediment. Fe^2^⁺ compresses the double electric layer, reducing repulsive forces between particles and promoting particle aggregation, while the ·OH and SO₄⁻· radicals generated through persulfate activation oxidize and disrupt the EPS structure, releasing a substantial amount of bound water. This mechanism is validated by the observed changes in EPS content, zeta potential, compression coefficient, EPR and microstructure.

#### 3.3.6 Cost-effectiveness and ecological considerations

While the process of acid washing, water rinsing, FeSO₄ modification, and co-application with Na₂S₂O₈ may appear complex, the overall cost-effectiveness is justified by the utilization of waste biomass, enhanced dewatering performance, and resource recovery. The use of Enteromorpha algae as a raw material not only reduces costs but also addresses the environmental issue of algal bloom management (Chen et al., 2022a; Brown et al., 2019). Additionally, the optimized use of chemicals minimizes costs while achieving maximum dewatering efficiency.

Regarding ecological risks, the acid used for washing is neutralized before disposal, and the biochar is rinsed to ensure no acidic residues are released. Na₂S₂O₈ is applied in low concentrations and fully activated to generate harmless byproducts. Environmental monitoring can be carried out to confirms that the treated sediment meets regulatory standards, posing minimal ecological risk.

## 4 Conclusion

This study successfully demonstrated the efficacy of modified Enteromorpha algae-derived biochar in enhancing the dewatering performance of marine sediment, particularly when activated with persulfate. Through optimized preparation conditions (700°C) and modification methods (2.5 mol/L FeSO₄), significantly improvements in dewatering performance were achieved. Specifically, after treatment with modified biochar-activated persulfate, the specific resistance to filtration (SRF) decreased by 73.4%, the net solid yield (Y_N_) increased by 105%, and the dewatering volume increased by 118%. Analysis of EPS revealed that the activated persulfate effectively degraded EPS in the sediment, releasing bound water and thus improving dewatering performance. Additionally, zeta potential measurements indicated that the introduction of Fe^2^⁺ significantly reduced the negative charge of the sediment, destabilizing the colloidal structure and promoting aggregation of sediment particles and water release. Changes in the compression coefficient further confirmed the optimized structural properties of the sediment, resulting in lower compressibility and higher porosity during dewatering. SEM observations also showed that the treated sediment had a more fragmented and porous microstructure, facilitating water removal.

This research not only provides an efficient and environmentally friendly technological solution for the dewatering of marine sediment but also offers a new perspective for the resourceful utilization of algal biomass and the ecological management of algal blooms. By transforming Enteromorpha algae into biochar and using it for sediment dewatering, we have realized the concept of “turning waste into treasure,” providing a scientific basis for marine environmental protection and resource recycling. Future research will further explore the large-scale application potential of this technology and optimize the process conditions to reduce treatment costs.

## Data Availability

The original contributions presented in the study are included in the article/supplementary material, further inquiries can be directed to the corresponding authors.
